# Perturbing Enhancer Activity in Cancer Therapy

**DOI:** 10.3390/cancers11050634

**Published:** 2019-05-07

**Authors:** Feda H. Hamdan, Steven A. Johnsen

**Affiliations:** Gene Regulatory Mechanisms and Molecular Epigenetics Lab, Division of Gastroenterology and Hepatology, Mayo Clinic, Rochester, MN 55905, USA; Hamdan.Feda@mayo.edu

**Keywords:** enhancers, BET inhibitors, CDK7 inhibitors, HDAC inhibitors, transcription factors, eRNAs, cancer

## Abstract

Tight regulation of gene transcription is essential for normal development, tissue homeostasis, and disease-free survival. Enhancers are distal regulatory elements in the genome that provide specificity to gene expression programs and are frequently misregulated in cancer. Recent studies examined various enhancer-driven malignant dependencies and identified different approaches to specifically target these programs. In this review, we describe numerous features that make enhancers good transcriptional targets in cancer therapy and discuss different approaches to overcome enhancer perturbation. Interestingly, a number of approved therapeutic agents, such as cyclosporine, steroid hormones, and thiazolidinediones, actually function by affecting enhancer landscapes by directly targeting very specific transcription factor programs. More recently, a broader approach to targeting deregulated enhancer programs has been achieved via Bromodomain and Extraterminal (BET) inhibition or perturbation of transcription-related cyclin-dependent kinases (CDK). One challenge to enhancer-targeted therapy is proper patient stratification. We suggest that monitoring of enhancer RNA (eRNA) expression may serve as a unique biomarker of enhancer activity that can help to predict and monitor responsiveness to enhancer-targeted therapies. A more thorough investigation of cancer-specific enhancers and the underlying mechanisms of deregulation will pave the road for an effective utilization of enhancer modulators in a precision oncology approach to cancer treatment.

## 1. Introduction

Cancer is a disease of aberrant transcription which is dependent on mechanisms enabling deregulated gene expression [1,2,3]. Enhancers are short genomic elements or clusters of elements, which are bound by tissue- or cell type-specific transcription factors (TFs) that activate target gene transcription in a distal and autonomous manner [4]. Soon after their discovery, enhancers were reported to drive differential transcriptional regulation in a more diverse and versatile manner compared to transcriptional regulation occurring (primarily) at proximal promoter regions [5]. Accordingly, it is not surprising that misregulation of these transcriptional hubs was linked to various diseases, including cancer [6,7,8]. For example, a chromosomal rearrangement in acute myeloid leukemia (AML) was found to bring an enhancer into the proximity of the oncogenic *MDS1* and *EVI1 complex* locus (*MECOM*), precipitating the malignancy [9]. The amplification of enhancers was also shown to play a role in the pathophysiology of prostate cancer and neuroblastoma [10,11]. Furthermore, hijacking of enhancers led to the activation of the oncogenic *Growth Factor Independent 1* family in medulloblastoma [12]. Additionally, reprogramming of the enhancer landscape in pancreatic cancer was reported to play a significant role in promoting a more aggressive phenotype [13,14,15]. Moreover, enhancers were implicated in therapy resistance in leukemia [16]. Accordingly, the eminent implication of enhancers in diseases led to the development of the term “enhanceropathies” and enhancer biology has become a focal point of interest when investigating novel therapeutic targets in cancer [17]. In this report, we review recent studies supporting the rationale of targeting enhancers in cancer. Additionally, we summarize the reported use of enhancer modulators in different cancer types. Finally, we discuss the challenges facing the use of enhancer modulators in the clinical setting.

## 2. Targeting Transcription Factor-Related Programs in Cancer

Sequence-specific binding of transcription factors (TF) underlies the selective activation of enhancers in different systems [18]. TFs provide a high degree of specificity in gene regulation by binding to their cognate DNA sequences across the genome to activate (or repress) transcription via recruitment of various co-activators, such as chromatin remodeling proteins and histone modifying enzymes [19,20]. Certain TFs have been identified to be lineage-specific and drive the differentiation of certain cellular states through the activation of different enhancer repertoires [19,21]. Moreover, it was reported that certain TFs, including the majority of tissue-specific TFs, display a larger number of binding sites at distal enhancers compared to proximal promoters [22]. Accordingly, agents specifically targeting the function of such transcription factors will, in turn, perturb the activity of the select set of enhancers controlled by the given TF.

Notably, direct manipulation of individual enhancer activity has recently been achieved via gene editing approaches such as CRISPR-Cas9. For example, fetal hemoglobin was effectively induced by disrupting the binding site of the TF GATA binding protein 1 (GATA1) at the upstream enhancer of the fetal hemoglobin repressor BAF chromatin remodeling complex subunit BCL11A (*BCL11A*) [23]. Additionally, using catalytically dead Cas9-Krüppel-associated box domain (dCas9-KRAB) to silence various enhancer constituents of the oncogenic sphingosine kinase 1 (*SPHK1*) led to a decrease in its levels, which was associated with attenuated proliferation and migration in hepatocellular carcinoma [24,25]. Recruitment of a trans-enhancer by a customized dCas9 was more recently shown to activate target gene transcription in various cancer cell lines [26]. While these approaches illustrate examples of direct manipulation of enhancers in certain contexts, they have limited applications as therapeutic options due to various reasons. Firstly, the lack of effective, safe and specific delivery options of the Cas9 system remains a major challenge in applying these machineries in therapy. One of the novel approaches currently under study to deliver the Cas9 system includes multistage delivery nanoparticle (MDNP), which still requires further validation [27]. Additionally, the use of permanent gene editing in humans faces numerous ethical complications. Most importantly, these approaches can only affect a single target, which is rarely sufficient to combat highly plastic malignancies. Furthermore, the ramifications of permanent changes in the genome sequence are poorly understood and may lead to irreversible adverse events.

While genetic manipulation of enhancer function remains challenging, an established clinically relevant approach to modulate enhancer activity is the perturbation or activation of the sequence-specific transcription factor(s) that are required for enhancer function. A primary example of such targeting is the perturbation or activation of steroid hormone receptors in various cancers, such as breast cancer [28,29,30], prostate cancer [31,32,33], and lymphomas [34,35]. For example, 70% of breast cancers are estrogen receptor-positive (ER+) and are, at least initially, highly responsive to endocrine therapy [36]. Estrogen receptor-alpha (ERα) is a master transcription factor in breast cancer which can be activated by estradiol. Estrogen binding to the ligand-binding domain of ERα leads to conformational changes which promote dimerization, subsequent binding to specific targets in the genome called estrogen response elements (EREs), and recruitment of co-activator proteins [37]. When ERα localization was investigated throughout the genome, it was quickly recognized that it rarely binds to promoter regions, but rather shows a tendency to localize to enhancer regions [38]. Interestingly, ERα was recently reported to nucleate phase-separated condensates at highly active enhancers [39], thereby promoting transcription at these extremely active hubs [40]. Thus, while endocrine therapy has been a central approach for treating a large number of breast cancer patients for over four decades, it has only recently been appreciated that the main molecular mechanisms by which tamoxifen and similar steroid hormone receptor antagonists exert their effects is by the modulation of enhancer activity. This principle is also applicable to other steroid hormone receptors, where (positively or negatively) targeting the enhancer function of the androgen receptor (AR) [41,42,43] or glucocorticoid receptor [44] has been shown to be very effective in other malignancies.

Other therapeutic agents that are used for other indications, such as the insulin sensitizing thiazolidinediones, also modulate the enhancer landscape by acting as agonists for the nuclear receptor Peroxisome Proliferator-Activated Receptor-γ (PPARG) [45]. In this case, treatment with rosiglitazone leads to the selective activation of PPARG-occupied enhancers. While thiazolidinediones were reported to have an inhibitory proliferative effect in hepatocellular and esophageal cancers, enhancer modulation by glitazones in cancer is still not very well studied [46,47,48]. Interestingly, a retrospective study observed a significant negative correlation of administration of thiazolidinediones and colorectal cancer, suggesting these agonists may have a protective role in preventing cancer [49]. Accordingly, those drugs which were originally developed for other indications may have a potential unlocked utility in some cancers. However, further investigation is needed to confirm the efficacy of these agents in this context.

While targeting nuclear receptors is one of the best examples of how directly perturbing transcription factor activity can be achieved, other frequently utilized therapeutic agents have a similar mechanism of action. For example, calcineurin inhibitors, which attenuate the calcium-dependent translocation of the Nuclear Factor of Activated T cells (NFAT), were shown to have growth inhibitory effects in various types of cancer, such as hepatocellular carcinoma, melanoma, and retinoblastoma [50,51,52]. NFAT was shown to elicit its effects at enhancer regions in blood vessel maturation [53] and function together with STAT3 at enhancers downstream of KRAS signaling in pancreatic cancer [54]. Thus, the use of calcineurin inhibitors will directly impact the activity of NFAT-driven enhancer programs and can be a promising approach in cancer therapy, especially in cases such as breast and pancreatic cancer where the NFAT pathway has been shown to be activated [45,55]. Conversely, cyclosporine has been implicated in increased risk for squamous cell carcinoma due to an increase of Activating Transcription Factor 3 (ATF3), which suppresses p53-induced senescence [56]. Thus, the context-specific effects of this drug should be closely considered before harnessing its enhancer-perturbing activity. Another approach to modulate the effects of a transcription factor can be by targeting its stability. For example, the Hypoxia Inducible Factor Alpha subunit (HIF1A) is a transcription factor that is known for its role in mediating the hypoxic response and it was shown to correlate with poorer prognosis in various types of cancer [57,58]. Enhancers also underlie the activity of HIF1A in modulating target gene expression [15,59,60,61]. Interestingly, topoisomerase I inhibitors were observed to inhibit the translation of HIF1A and may therefore function in part by this mechanism in the case of cancers where this factor plays a significant role [62].

As these drugs target particular transcriptional programs driven by the activity of a highly specific group of enhancers, their effectiveness has shed light on the benefits of targeting deregulated enhancer programs in specific disease contexts. Accordingly, targeting deregulated enhancer activity is, in fact, already an established paradigm and mainstay in the clinical treatment of cancer.

## 3. Perturbing Enhancer Activity by Therapeutic Agents and Inhibitors

**Figure 1:**
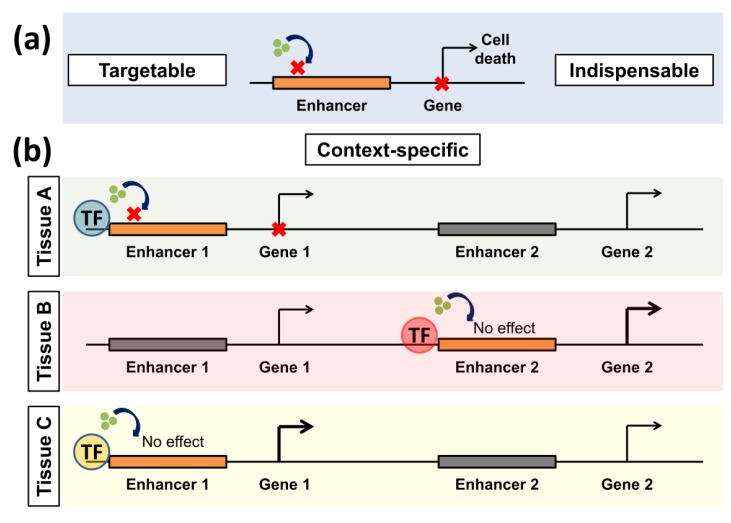
Positive features rendering enhancers good transcriptional targets. (**a**) Enhancers can be pharmacologically manipulated using different small molecule inhibitors (indicated by green dots). They are also indispensable for cancer cells as they activate important oncogenes. (**b**) Enhancers are also context-specific. In this example, enhancers are activated by different transcription factors (TFs) in various tissues (A–C). The same inhibitor affects only a specific enhancer in a tissue if it is activated by a certain TF. Thus the illustrated inhibitor only affects Enhancer 1 in tissue A but not C. It also has no effect on the other active enhancers in tissue B. Gray enhancers are inactive, while orange ones are active. Bold arrows represent active transcription.

Expansion of our understanding of complex processes controlling gene regulation has uncovered numerous novel targets that can (potentially) be therapeutically modulated in cancer. However, such exponential growth in knowledge rendered the task of identifying and investing in a select few effective and relatively safe transcriptional targets immensely challenging. An ideal transcriptional target in cancer therapy would necessarily exhibit certain attributes which can lead to a perceptible change in the quality of life, prognosis, and the therapeutic management of patients. To achieve this, such a target must be amenable to inhibition, preferably by small molecules that have good bioavailability at the target site with an acceptable half-life (i.e., good pharmacokinetic and pharmacodynamics properties). Importantly, the ideal target should also have a specificity that spares non-transformed cells, thereby avoiding or minimizing any potential unwanted side effects caused by perturbations of normal cellular processes in healthy tissue (adverse or severe adverse events). Additionally, this target should be indispensable (non-redundant) to cancer cells, rendering them highly dependent on such a target. Finally, this dependence should ideally be shared by all or a high percentage of the malignant cell population in a given patient. As some enhancers or enhancer programs exhibit these characteristics, they provide promising transcriptional targets (Figure 1).

### 3.1. Enhancers Are Context-Specific and Indispensable for Cancer Cells

One of the major characteristics of a subgroup of enhancers is context-specificity. In general, enhancers direct lineage-specific transcriptional programs in a more predominant manner compared to promoters [63]. Around half of the enhancers identified in different tissues including brain, heart, ovaries, and placenta were tissue-specific [64]. Consistently, enhancers were the most distinctive identifying feature of tissue of origin upon analysis of hundreds of patient samples and human cell lines [65]. Interestingly, not only is there a distinct pattern of enhancer activation through tissues, but it was also observed that the interaction between active enhancers and their target genes are highly variable in various tissues as well [66]. Notably, interactions between enhancers and their target genes are variable in different systems and show even more tissue-specificity than differential activation of enhancers themselves, thus providing an additional layer of complexity.

In addition to having tissue-specificity, in order to be safely targetable, enhancers must exhibit specific activation in malignant cells compared to the tissue of origin. In this case, this activation should represent a new cancer cell-specific dependence that is not shared by healthy cells from the same tissue. Consistently, aberrant hypermethylation of enhancers in renal cancer cells led them to be more sensitive to the DNA methyltransferse (DNMT) inhibitor, decitabine, compared to healthy renal tissue [67]. More specifically, loss of the X-linked gene *Lysine Demethylase 6A* (KDM6A) resulted in a gender-specific aberrant activation of a set of enhancers leading to an aggressive phenotype of pancreatic cancer [68]. Importantly, inactivation of enhancers through Bromodomain and Extraterminal (BET) inhibitor treatment was effective in targeting this specific subtype of pancreatic cancer compared to other subtypes. This shows that enhancer specificity can extend to certain subtypes of cancer, adding a further layer of specificity and potentially increasing safety in targeting those elements. 

### 3.2. Activity of Enhancers Can Be Pharmacologically Perturbed

Enhancers were shown to be specifically targetable by various small molecule inhibitors. Preferential dependence of enhancers on Bromodomain and Extraterminal (BET) proteins has been consistently reported in various cancer types such as lymphoma [69], ovarian cancer [70], breast cancer [71,72], pancreatic cancer [68,73], leukemia [74], multiple myeloma, and glioblastoma [74,75]. Other modulators with reported efficacy on enhancers include inhibitors of the transcriptional cyclin dependent kinases-7 (CDK7) and -9 (CDK9).

#### 3.2.1. Epigenetic Modulators

Epigenetic regulation enables cells to control gene transcription in a manner complementary to sequence-specific transcription factor-based mechanisms. Such regulatory mechanisms include post-translational modification of histones, DNA methylation, nucleosome remodeling, and non-coding RNAs (ncRNAs) [76]. Histone marks do not act independently of one another, but rather cooperate to control gene transcription in what is referred to as “histone crosstalk” [77]. Eminent factors in the epigenetic machinery are so-called epigenetic “readers”, which recognize specific histone marks and recruit additional effectors [78]. An extensively studied example is the BET family of proteins, which each contain two bromodomains that can interact with acetylated lysine residues on target proteins via a hydrophobic pocket, thereby endowing BET proteins with the ability to recognize acetyl marks on chromatin [79]. JQ1 is a thienodiazepine that displaces the BET family member Bromodomain containing 4 (BRD4) from acetylated lysines by forming hydrogen bonds with a conserved asparagine residue that is situated in the hydrophobic pocket of BRD4 [80]. Many other BET inhibitors have also been developed, such as I-BET151, I-BET762, and OTX-015 [80,81,82]. In Diffuse Large B-Cell Lymphoma, BET inhibitors showed a marked effect on a subset of enhancers, termed super enhancers, that are highly enriched with BRD4 [69]. Super enhancers (SEs) were first identified as major drivers of gene expression that are highly enriched with transcription factor binding sites and include clusters of highly active distal regulatory elements [75,83]. SEs were observed to drive lineage-specific programs in various systems, such as epithelial differentiation, mesenchymal multipotency, and estrogen-dependent mammary gland malignancy, and showed sensitivity to BET inhibition [75,84,85,86]. Consistently, treating ovarian cancer cells with BET inhibitors diminished the activity of a super enhancer activating the chemoresistance-related aldehyde dehydrogenase and led to increased sensitivity to cisplatin treatment [70]. Additionally, treating various sensitive colorectal cancer cells with BET inhibitors attenuated the activity of enhancers gained in cancer compared to normal crypts [87]. While different super enhancer programs were identified in various subtypes of ependymomas, a general sensitivity to BET inhibition was reported in ependymoma cells [88]. The same pattern of activation of distinct BET-dependent super enhancers was also reported in chronic lymphocytic leukemia [89]. Enhancers driving the transcription of receptor tyrosine kinases that play a fundamental role in gastrointestinal stromal tumors have also shown dependence on BET family members [90].

Other important members of the epigenetic machinery include writers which act by selectively adding chemical moieties to a specific histone residue. Histone acetyltransferases (HATs), such as p300 and CREB-binding protein (CBP), transfer an acetyl group from acetyl-CoA to histone tails [91]. Inhibiting HATs in pancreatic cancer affected the activation of a certain subset of enhancers that are enriched by the Wnt-signaling transcription factor, Transcription Factor 7 Like 2 (TCF7L2) [86]. Furthermore, the *Polycomb* Repressive Complex-1 (PRC1) and -2 (PRC2) are extensively studied complexes which mediate monoubiquitination of H2A at lysine 119 (H2Aub1) and tri-methylation of histone 3 lysine 27 (H3K27me3), respectively [92]. As H2K27me3 is a histone mark which is highly associated with gene inactivation [93,94], targeting constituents of the PRC complex led to a specific de-depression of a specific set of enhancers in leukemia [95]. Consistently, targeting the catalytic subunit of the PRC2 complex, *Enhancer of Zeste* Homolog 2 (EZH2), led to the de-depression of enhancers controlling the pro-apoptotic B cell lymphoma-2 like 11 (*BIM*), thereby mediating apoptosis in breast cancer cells [96].

Other classes of important epigenetic factors include “erasers”, enzymes that remove histone marks [97]. This includes histone deacetylases (HDACs), which mediate the removal of lysine acetylation and consist of multiple classes that can also mediate de-acetylation of non-histone proteins [98]. HDAC inhibitors were found to affect the enhancer landscape in colorectal, pancreatic, and breast cancer [96,99,100]. While methylation was previously considered to be an irreversible modification, Lysine-Specific Demethylase 1 (LSD1, also called KDM1A) was identified in 2002 as a selective mediator of the de-methylation of histone 3 lysine 4 [101,102]. Mono-methylation and tri-methylation of histone 3 lysine 4 (H3K4me1 and H3K4me3) are known marks for gene activation at distal and proximal regulatory regions, respectively [76,103]. LSD1 inhibitors affect a specific subset of enhancers controlling differentiation in acute myeloid leukemia by disrupting their interaction with the SNAG-domain transcription repressor GFI1 [104]. LSD1 has also been shown to influence enhancer activity in a number of other systems including embryonic stem cell differentiation [105], androgen receptor function in prostate cancer [106,107], and ERα activity in breast cancer [108]. Altogether, epigenetic modulators provide a variety of targets which can be manipulated to modulate the cancer-specific enhancer landscape and affect transcriptional programs. While current research is largely focused on BET inhibitors and their role in affecting enhancers, many other epigenetic inhibitors may potentially also be used in the context of enhancer activity manipulation as further mechanisms and contexts are better defined.

#### 3.2.2. Cyclin-Dependent Kinase Inhibitors

The recruitment of RNA Polymerase II (RNA Pol II) to the proximal promoter enables the initiation of transcription, which is signified by the phosphorylation of serine 5 within the heptapeptide repeats of the carboxy-terminal domain (CTD) and the subsequent capping of nascent RNA [109,110]. Pol II is frequently temporarily paused by the Negative Elongation Factor (NELF) and DRB-sensitivity Inducing Factor (DSIF) within the first 100 nucleotides after the transcription start site (TSS) [109,111,112]. Thereby, this promoter proximal pausing has been regarded as a crucial rate-limiting step for gene transcription in metazoans [113]. To proceed to productive elongation, the Positive Transcription Elongation Factor-b (P-TEFb) phosphorylates Pol II at serine 2 of the CTD as well as components of both the NELF and DSIF complexes [114,115] via its catalytic subunit Cyclin-Dependent Kinase-9 (CDK9) bound to the cognate cyclin T1 [116]. These phosphorylation events release Pol II from promoter proximal pausing and allow transcription elongation until polyadenylation sequences are transcribed, which leads to the cleavage and subsequent polyadenylation of mRNAs [117]. Notably, inhibition of CDK9 not only attenuates transcription elongation of the pre-mRNA, but also decreases eRNA production at enhancer regions [118]. Consistent with complementary functions in controlling enhancer function, the combination of CDK9 inhibition along with BET inhibitors has shown enhanced effects in both AML [119] and malignant rhabdoid tumor cells [120]. As a monotherapy, CDK9 inhibitors were observed to highly inhibit the expression of genes associated with super enhancers, such as *MYC* [121]. In chordoma, a highly aggressive tumor of the bone, inhibition of CDK9 and CDK7 has been reported to be highly effective [122].

As part of the TFIIH complex, CDK7 plays an important role in gene transcription via phosphorylation of the Pol II CTD at Ser5 [123,124]. It was reported that phosphorylation of the CTD by CDK7 leads to the dissociation of the CTD with DNA and the initiation of transcription [125]. CDK7 also plays a dual role in controlling cell cycle progression by phosphorylating and activating CDK1 and CDK2 [126]. Inhibition of CDK7 by the covalent inhibitor, THZ1, was found to be highly toxic to cancer cells, presumably by specific inactivation of super enhancers [127]. Indeed, super enhancers controlling the *MYCN* proto-oncogene were selectively inactivated by THZ1 in neuroblastoma [128]. Interestingly, several reports followed observing a selective perturbation of super enhancer programs by inhibition of CDK7 in small cell lung cancer [129], triple-negative breast cancer [130], ovarian cancer [131], esophageal carcinoma [132], melanoma [133], gliobastoma multiforme [134], and pancreatic cancer [135]. However, inhibition of CDK7 was reported to increase characteristics associated with metastasis in colorectal cancer cells [136]. THZ1 was found to attenuate the normal transition of the various stages of transcription starting from initiation into elongation [137]. Conversely, while THZ1 appears to preferentially affect super enhancer-associated genes, this effect does not appear to be due to altered RNA Pol II activity directly at the enhancers themselves [138]. Moreover, a more recent report suggests that the effects of THZ1 on super enhancer-associated genes may, in fact, be due to the off-target inhibition of CDK12 and CDK13, rather than CDK7 [139]. Thus, further studies to understand the exact mechanism of selective attenuation of super enhancer activation are necessary before the use of CDK7 (or CDK12/13) inhibitors can be precisely tested in the clinical setting on a mechanistic basis. Currently, two early-phase clinical studies are ongoing to investigate the use of CDK7 inhibitors in patients with advanced solid malignancies (NCT03363893, NCT03134638).

**Figure 2:**
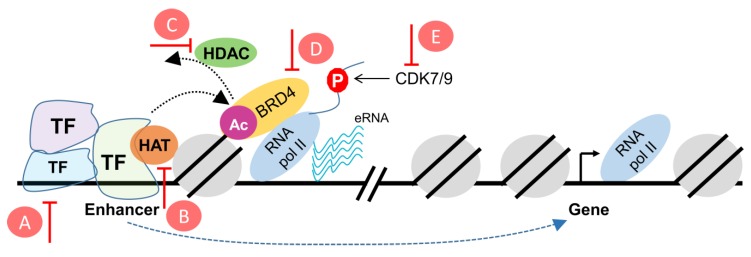
Schematic representation of putative targets to reprogram the enhancer landscape in cancer. (**A**) modulators of transcription factors, (**B**) HAT inhibitors, (**C**) HDAC inhibitors, (**D**) BET inhibitors, (**E**) CDK7/9 inhibitors. HAT: Histone acetyltransferase; HDAC: Histone deacetylase; BET: Bromodomain and extraterminal; CDK: Cyclin-dependent kinases.

In addition to the previously mentioned regulators, the Mediator complex plays a crucial role in transcriptional regulation [140,141]. Mediator is a large multi-subunit complex that plays a crucial role in the assembly and activation of the pre-initiation complex (PIC) by forming a bridge between various sequence-specific transcription factors and components of the PIC [142]. In addition to its important role at gene promoters, Mediator is reported to connect initiating promoters with active distal enhancers through chromatin loop formation [143]. The first evidence of chromatin loop formation where a distal region affected the transcription of a target gene promoter was reported in 1984 by Dunn et al. [144] in bacteria. Approximately 20 years later, cohesin, which also plays a central role in sister-chromatid adhesion, was revealed to orchestrate the formation of DNA loops with the help of the insulator, CCTC-Binding Factor (CTCF), and the cohesin loader, Nipped-B-Like (NIPBL) [145,146,147]. Mediator was found to bind cohesin and NIPBL to bring active enhancers and promoters into close proximity [143]. As Mediator is composed of approximately 30 subunits, it can have different conformations [148]. One conformation includes the kinase module containing cyclin-dependent kinase 8 (CDK8), which does not appear to directly phosphorylate the RNA Pol II CTD, but was shown to have more preference toward affecting active enhancers [149]. In contrast to BET and CDK7 inhibition, CDK8 inhibitors have been reported to have an activating effect on super enhancers in leukemic cells [150]. Interestingly, given the proposed dose-dependent function of these enhancers, leukemic cells were still impaired in their growth following treatment with a CDK8 inhibitor. Additionally, CDK8 inhibitors were reported to be crucial in mediating the transcriptional effects of HIF1A [151] as well as beta-catenin in colorectal cancer [152]. Accordingly, CDK8 inhibition can lead to changes in the enhancer landscape by various mechanisms. A number of the described targets which modulate enhancer activity are illustrated and summarized in Figure 2.

## 4. Challenges Facing the Utility of Enhancer Modulators in the Clinical Setting

While targeting transcriptional enhancers is still under investigation, compensatory resistance mechanisms upon inhibition of active enhancers have already been described. For example, the BET inhibitor JQ1 was reported to induce resistance mediated by transcriptional activation in bromodomain-independent pathways in castration-resistant prostate cancer [153]. Interestingly, this resistance uncovered an alternative dependency on CDK9-mediated activation of androgen receptor signaling. In pancreatic cancer, upregulation of the *GLI Family Zinc Finger 2* (*GLI2*) was found to enable resistance to BET inhibition [154]. In leukemia, resistance to BET inhibition was partly caused by an increase in Wnt-signaling pathway activity [155,156]. Interestingly, while MAPK/ERK kinase inhibition (MEKi) sensitized colorectal cancer cells to BET inhibition, BET inhibitors sensitized MEKi-resistant cells in breast cancer [71,157]. Similarly, inhibitors of the Nuclear Factor Kappa-light-chain-enhancer of activated B cells (NFKB) pathway led to sensitization to BET inhibitors in uveal melanoma [158]. Coupling the inhibition of BET-dependent enhancers with targeting of other transcriptome regulating axes such as E2F-dependent promoters was suggested as an effective approach to target gene transcription in multiple myeloma [159]. Consequently, it is not unlikely that using enhancer modulators will reveal various challenges for their use in the clinical setting. These will include the development of resistance in addition to the difficulty in predicting responsiveness.

Notably, while the context-specific properties of enhancers can be leveraged in precise targeting of particular enhancer programs, it will also lead to inconsistent performances in various cancer entities. Accordingly, it is not surprising that enhancer modulators may show highly promising effects in one cancer type and fail in another. For example, inhibition of EZH2 was reported to be effective in lymphomas where aberrant histone methylation is caused by mutation in this particular regulator [160]. On the other hand, the depletion of EZH2 activity caused by histone 3.3 (H3.3) mutations in diffuse intrinsic pontine gliomas are major precipitants of the disease [161]. Additionally, malignant peripheral nerve sheath tumor (MPNST) is largely caused by mutations in the Ras signaling suppressor neurofibromin 1 (NF1) leading to activation of Ras signaling and usually accompanied by mutations in p53 and the PRC2 complex members, SUZ12 and EED, which lead to a loss of H3K27me3 [162,163,164]. Thus, treatment with EZH2 inhibitors in certain contexts (such as neurofibromatosis type 1) may actually exacerbate or precipitate tumorigenesis or tumor progression. This apparent discrepancy is very likely due to the importance of a balanced level of histone modifications to ensure homeostasis, as well as the signaling, genetic, and epigenetic context-dependency. Another example includes the use of HDAC inhibitors in various types of cancer. Multiple HDAC inhibitors have been approved by the United States Food and Drug Administration (FDA) for use in indications such as relapsed and refractory cutaneous T-cell lymphoma, peripheral T cell lymphoma, and multiple myeloma [165]. Conversely, clinical trials showed limited effects of HDAC inhibitors in other cancer types, such as head and neck [166], breast [167], ovarian [168], and pancreatic cancer [169]. However, better effects were observed in some cases when HDAC inhibitors were used in combination with other therapies such as BET inhibitors [73], chemoradiation [170], and VEGF inhibitors [171]. These observations underscore the complexity of the clinical use of enhancer modulators and the major challenges facing these agents. They also support the need for a deeper understanding of aberrant enhancer programming in different malignancies and how this relates to the effectiveness of enhancer-targeting therapies.

**Figure 3:**
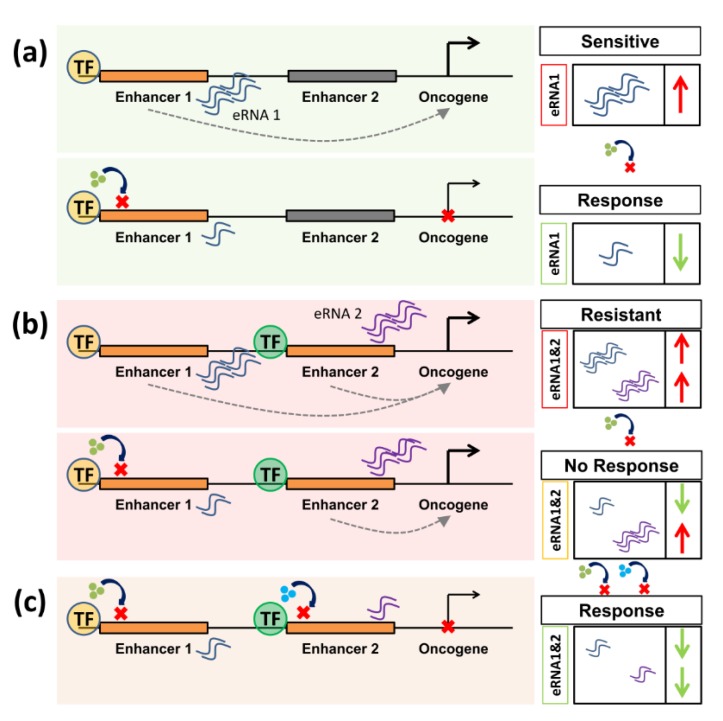
Enhancer RNAs (eRNAs) are putative biomarkers for responsiveness and resistance in perturbation of enhancer activity. (**a**) In a responsive context, inhibiting an enhancer leads to a decrease in the activity of oncogenic target genes. In this case, high levels of eRNA can predict responsiveness to a specific inhibitor by providing a direct readout of enhancer activity. (**b**) In case of resistance, compensating mechanisms such as the activation of a different enhancer program can occur. Thereby, high levels of different eRNAs can predict resistance to a certain therapy. (**c**) To re-sensitize cells, compensatory mechanisms should also be targeted to ensure therapeutic success.

As described above, a major challenge in the clinical utilization of enhancer-targeting approaches to cancer therapy is the identification of potentially responsive patients for proper stratification. A breakthrough in this may be the use of so-called “eRNAs” (enhancer ribonucleic acids) as clinical pathologic indicators of active enhancer programs in tumor cells. Although distal intergenic enhancer regions were not previously thought to be transcribed, it has since been shown that transcription occurs at these regions in contradiction to the general trends of energy conservation inside the cell [172]. The functions and mechanisms of the resulting eRNA products are still not fully elucidated [173]. However, eRNAs appear to provide excellent markers of enhancer activity that likely outperform information on the occupancy of transcription activators or histone marks [42]. Functionally, eRNAs were reported to augment gene transcription as their knockdown led to decreased target gene transcription [174,175]. Furthermore, chromatin loop formation and eRNA production were reported to precede transcription of the mRNA [176] and, more recently, eRNAs were reported to promote the formation of phase-separated nuclear interchromatin granules associated with actively transcribed genes [39]. Irrespective of their function, we suggest that these products may provide particularly useful clinical markers for predicting and monitoring therapeutic responsiveness and resistance (Figure 3).

This is of particular interest as eRNAs were reported to be highly enriched at tissue-specific enhancers [177]. Indeed, the eRNA *CCAT1* was proposed as a therapeutic biomarker that can predict responsiveness to BET inhibition [178]. Interestingly, an enhancer identified in prostate cancer, which is associated with the gene encoding prostate-specific antigen (PSA) and the resulting eRNA, was shown to play a central role in controlling gene transcription in prostate cancer cells [179]. Additionally, Kaczkowski et al. [180] identified 90 eRNAs that are generally upregulated in cancer cells upon screening over 200 cell lines and approximately 300 primary samples. Identification of eRNAs has been made feasible due to the development of techniques such as global run-on sequencing (GRO-seq) [181], transient transcriptome sequencing (TT-seq) [182], precision nuclear run-on sequencing (PRO-seq) [183], and, more recently, chromatin run-on and sequencing (ChRO-seq) [184]. Notably, length-extension ChRO-seq enables the detection of nascent RNA from tissue samples that were stored for longer periods of up to 30 years. Thus, current technologies allow us to more easily identify eRNAs from patient samples irrespective of sample quality, further enabling a potential utilization of eRNAs as enhancer biomarkers. Importantly, manipulation of these shortly-lived transcripts by cell-permeable synthetic antisense oligonucleotides (ASOs) [185] can also affect target gene transcription and may be an effective mechanism of perturbing enhancer activity [186].

Notably, other technical challenges face the elucidation of the mechanisms of enhancer functions and their targeting. Enhancers are usually identified using highly complex bioinformatic analyses that are not always accessible to clinicians and scientists alike. Identification of important enhancers has also been accompanied by the emergence of different subclasses of enhancers. Since the recent identification of super enhancers in 2013 [6,75], approximately 300 scientific papers discussing this subclass have been published. Another class of enhancers called “stretch enhancers” are sometimes used interchangeably with super enhancers [187,188]. This comparison is, however, somewhat inaccurate as studies indicate that stretch enhancers only meet the requirement of spanning long stretches of DNA but, unlike super enhancers, are not necessarily rich with transcription factors or cell-specific [189]. An additional subclass includes “shadow enhancers”, which are a group of “secondary” enhancers that are superfluous and redundant to an active enhancer, thereby ensuring the precision of gene transcriptional regulation [190]. Such a concept, which was first identified in *Drosophila*, has also been reported in mammals [191]. This led to the sometimes imprecise use of the term “shadow enhancers” to describe typical enhancers, which are not necessarily supportive of other enhancers and may play decisive roles in tissue- and cancer-specific gene regulation in their own right. A clearer definition of these new classifications will significantly help in a better and more precise understanding of enhancer activity and its modulation.

Another crucial hurdle facing the investigation of the role of enhancers in transcriptional activation is the complexity of defining the target genes of each enhancer. In the cell, targets of enhancers are not necessarily in close linear (genomic) proximity and can be separated by many unaffected genes [192]. As previously reported, interactions between enhancers and their target genes are variable in different systems and can show more tissue specificity than differential activation of enhancers themselves [66]. Chromatin conformation capture assays to detect interactions between cis-regulatory elements were first established in 2002 and have been followed by many techniques that extended our knowledge about the interactions between enhancers and their target promoters [193,194,195,196]. As these techniques are difficult to perform in patient samples and are generally not very cost-effective, identification of target genes in a concise manner in patient samples remains challenging.

## 5. Conclusions and Future Directions

Aberrant transcriptional regulation is one of the characteristics of malignancy which can be most efficiently and specifically manipulated through enhancer elements. A greater breadth of knowledge about activated enhancers or super enhancers, interconnected with dependencies and biomarkers, may play a significant role in the optimization of therapy for patients suffering from cancer and other diseases. In conclusion, enhancers exhibit many attributes of an ideal transcriptional target and are highly promising to be leveraged in cancer therapy and management. This is due to the fact that they are targetable, specific, and indispensable. They also frequently produce products (eRNA) that may potentially be utilized as predicative biomarkers and/or for monitoring therapeutic effectiveness. However, the targeting of compensatory mechanisms in response to their modulation should be considered as well. Given the clear significance of targeting enhancers in cancer, more studies are needed to further expand the currently available agents modulating the activity of these extremely important transcription targets.

As newer technologies to modulate targets become available in the future, our abilities to manipulate gene expression programs in cancer would be exponentially expanded. Our group and others [14,15] uncovered a role of the transcription factor deltaNp63 as a major driver of a more aggressive subtype of pancreatic cancer (squamous subtype) by activating a squamous enhancer/super enhancer program. The fact that deltaNp63 overexpression was sufficient to activate the squamous program suggests that this transcription factor may be an Achilles heel in this particular subtype [14]. DeltaNp63 was reported to be highly expressed in cancers and molecular subtypes of cancer that are squamous or basal in their nature, such as breast, head and neck, lung, and esophageal carcinoma [197,198,199,200]. We suggest that these findings may serve as a basis and rationale for the next step in precision oncology and the design of future basket trials where the effect of targeting deltaNp63 and its downstream activated enhancers can be investigated in malignancies that are divergent in origin but similar in the enhancer programs that drive their identity and aggressiveness.
